# Supplementary material for: Normalized Indices Derived from Visceral Adipose Mass Assessed by Magnetic Resonance Imaging and Their Correlation with Markers for Insulin Resistance and Prediabetes
Source: Nutrients. 2020 Jul 11;12(7):2064. doi: 10.3390/nu12072064 (PMC7400828; doi:10.3390/nu12072064)
Supplement: Supplementary file 1 [file nutrients-12-02064-s001.pdf]

Supplementary Data to the Manuscript

Normalized Indices Derived from Visceral Adipose Mass Assessed by Magnetic Resonance Imaging and Their Correlation with Markers for Insulin Resistance and Prediabetes

Jürgen Machann [1,2,3,*], Norbert Stefan [1,2,4], Robert Wagner [1,2,4], Andreas Fritsche [1,2,4], Jimmy D. Bell [5], Brandon Whitcher [5], Hans-Ulrich Häring [2], Andreas L. Birkenfeld [1,2,4], Konstantin Nikolaou [6], Fritz Schick [1,2,3] and E. Louise Thomas[5]

1 Institute for Diabetes Research and Metabolic Diseases (IDM) of the Helmholtz Center Munich at the University of Tübingen, 72076 Tübingen, Germany; norbert.stefan@med.uni-tuebingen.de (N.S.); Robert.wagner@med.uni-tuebingen.de (R.W.); andreas.fritsche@med.uni-tuebingen.de (A.F.); andreas.birkenfeld@med.uni-tuebingen.de (A.L.B.); fritz.schick@med.uni-tuebingen.de (F.S.)

2 German Center for Diabetes Research (DZD), 72076 Tübingen, Germany; haerinhu@icloud.com

3 Section on Experimental Radiology, Department of Diagnostic and Interventional Radiology, University Hospital Tübingen, Hoppe-Seyler-Str. 3, 72076 Tübingen, Germany

4 Department of Endocrinology, Diabetology and Nephrology, University Hospital Tübingen, 72076 Tübingen, Germany

5 Research Centre for Optimal Health, School of Life Sciences, University of Westminster, London W1W 6UW UK; J.Bell@westminster.ac.uk (J.D.B.); B.Whitcher@westminster.ac.uk (B.H.); L.Thomas3@westminster.ac.uk (E.L.T)

6 Department of Diagnostic and Interventional Radiology, University Hospital Tübingen, 72076 Tübingen, Germany; konstantin.nikolaou@med.uni-tuebingen.de

* Correspondence: juergen.machann@med.uni-tuebingen.de

Table S1

a) Anthropometric and metabolic data for the TDFS cohort including significance levels between females and males as well as difference of mean (Diff of Mean) and upper/lower 95% confidence intervals (CI)

	all		females		males		
	1295		801		494		
	mean±SD	range	mean±SD	range	mean±SD	range	p
age [years]	44.8±13.3	18-77	44.1±12.7	18-77	45.9±14.2	18-75	0.029
	Diff of Mean:	*1.8*	*Upper CI:*	*3.17*	*Lower CI:*	*0.17*	
height [cm]	170.7±8.9	148-198	165.9±6.3	148-185	178.6±6.6	159-198	< 0.0001
	Diff of Mean:	*12.7*	*Upper CI:*	*13.41*	*Lower CI:*	*11.96*	

BMI [kg/m²]	29.9±5.9	17.7-55.1	29.8±6.2	17.7-55.1	30.1±5.4	18.6-50.8	0.338
	Diff of Mean:	*0.3*	*Upper CI:*	*0.99*	*Lower CI:*	*-0.43*	
WHR	0.89±0.09	0.61-1.18	0.85±0.07	0.61-1.15	0.97±0.07	0.70-1.18	< 0.0001
	Diff of Mean:	*0.12*	*Upper CI:*	*0.133*	*Lower CI:*	*0.118*	
ISI Mats [a.u.]	9.8±6.1	0.9-38.1	10.2±6.1	1.2-38.1	9.2±6.0	0.9-32.9	0.0028
	Diff of Mean:	*-1.05*	*Upper CI:*	*-0.36*	*Lower CI:*	*-1.74*	
HbA1c [mmol/mol]	37.7±4.5	21.3-54.1	37.7±4.3	25.7-54.1	37.6±4.8	21.3-54.1	0.740
	Diff of Mean:	*0.07*	*Upper CI:*	*0.44*	*Lower CI:*	*-0.61*	

b) Anthropometric and metabolic data in UKBB study cohort including significance levels between females and males as well as difference of mean (Diff of Mean) and upper/lower 95% confidence intervals (CI)

	all		females		males		
	1295		801		494		
	mean±SD	range	mean±SD	range	mean±SD	range	p
age [years]	55.4 ± 7.5	40-70	54.7 ± 7.4	40-70	56.2 ± 7.6	40-70	<0.00001
	Diff of Mean:	*-1.46*	*Upper CI:*	*-1.75*	*Lower CI:*	*-1.17*	
height [cm]	169.1 ± 9.2	141-201	162.8 ± 6.2	141-195	176.0 ± 6.6	152-198	<0.00001
	Diff of Mean:	*-13.25*	*Upper CI:*	*-13.50*	*Lower CI:*	*-13.00*	
BMI [kg/m²]	26.7 ± 4.4	14.2-55.2	26.2 ± 4.7	14.2-55.2	27.1 ± 3.9	16.4-48.4	<0.00001
	Diff of Mean:	*-0.87*	*Upper CI:*	*-1.04*	*Lower CI:*	*-0.70*	
WHR	0.86 ± 0.08	0.62-1.20	0.81 ± 0.07	0.62-1.11	0.92 ± 0.06	0.73-1.15	<0.00001
	Diff of Mean:	*-0.11*	*Upper CI:*	*-0.113*	*Lower CI:*	*-0.108*	
HbA1c [mmol/mol]	35.1 ± 5.0	16.1-93.6	34.9 ± 4.7	21.8-91.1	35.3 ± 5.4	16.1-93.6	0.0002
	Diff of Mean:	*-0.40*	*Upper CI:*	*-0.04*	*Lower CI:*	*-0.01*	

Table S2

a) MR-derived parameters for the TDFS cohort including significance levels between females and males with difference of mean and upper/lower 95% confidence intervals (CI)

	all		females		males		
	mean±SD	range	mean±SD	range	mean±SD	range	p
TAT [l]	36.0±14.4	7.8-103.8	37.8±14.6	10.1-96.3	33.3±13.7	7.8-103.8	< 0.0001
	Diff of Mean:	*-4.5*	*Upper CI:*	*-2.90*	*Lower CI:*	*-6.13*	
TLT [l]	48.2±10.0	29.5-84.5	42.3±5.9	29.5-68.7	57.6±7.7	39.2-84.5	< 0.0001
	Diff of Mean:	*15.3*	*Upper CI:*	*16.08*	*Lower CI:*	*14.57*	
VAT [l]	3.8±2.4	0.3-13.9	2.9±1.7	0.3-10.9	5.5±2.6	0.4-13.9	< 0.0001
	Diff of Mean:	*2.60*	*Upper CI:*	*2.83*	*Lower CI:*	*2.37*	
VAT/m [l/m]	2.24±1.37	0.14-8.09	1.72±1.03	0.14-6.59	3.06±1.44	0.25-8.09	< 0.0001
	Diff of Mean:	*1.34*	*Upper CI:*	*1.47*	*Lower CI:*	*1.20*	
VAT/m² [l/m²]	1.30±0.78	0.08-4.74	1.04±0.63	0.08-4.07	1.72±0.81	0.14-4.74	< 0.0001
	Diff of Mean:	*0.68*	*Upper CI:*	*0.75*	*Lower CI:*	*0.60*	
VAT/m³ [l/m³]	0.76±0.45	0.05-2.78	0.63±0.39	0.05-2.56	0.97±0.46	0.08-2.78	< 0.0001
	Diff of Mean:	*0.34*	*Upper CI:*	*0.38*	*Lower CI:*	*0.29*	
%VAT	10.9±6.0	1.5-30.3	7.4±3.1	1.5-22.2	16.5±5.2	2.4-30.3	< 0.0001
	Diff of Mean:	*9.1*	*Upper CI:*	*9.6*	*Lower CI:*	*8.7*	
VAT/TLT	7.7±4.0	0.6-22.3	6.6±3.6	0.6-20.7	9.4±4.0	0.8-22.3	< 0.0001
	Diff of Mean:	*2.8*	*Upper CI:*	*3.2*	*Lower CI:*	*2.3*	
VAT/WEI	4.2±2.1	0.4-13.0	3.3±1.6	0.4-9.7	5.5±2.1	0.5-13.0	< 0.0001
	Diff of Mean:	*2.2*	*Upper CI:*	*2.4*	*Upper CI:*	*2.0*	

b) MR-derived parameters in UKBB study cohort including significance levels between females and males

	all		females		males		
	mean±SD	range	mean±SD	range	mean±SD	range	
TrunkAT [l]	10.7±4.5	0.93-32.6	10.7±4.7	0.9-32.6	10.8±4.5	1.0-32.6	0.529
	Diff of Mean:	-0.22	*Upper CI:*	-0.45	*Upper CI:*	0.009	

TMVol[#] [l]	10.3±2.6	3.8-21.4	8.4±1.2	3.8-14.8	12.5±1.8	5.2-21.4	$p < 0.0001$
	Diff of Mean:	-4.17	*Upper CI:*	-4.23	*Upper CI:*	-4.11	
VAT [l]	3.7±2.2	0.1-14.4	2.6±1.5	0.1-12.1	4.9±2.3	0.3-14.4	$p < 0.0001$
	Diff of Mean:	-2.25	*Upper CI:*	-2.33	*Upper CI:*	-2.18	
VAT/m [l/m]	2.18 ±1.27	0.07-8.72	1.62±0.93	0.07-7.5	2.77 ±1.31	0.14-8.72	$p < 0.0001$
	Diff of Mean:	-1.16	*Upper CI:*	-1.20	*Upper CI:*	-1.11	
VAT/m^2 [l/m^2]	1.28±0.72	0.05-5.49	1.00±0.58	0.05-4.61	1.58±0.75	0.08-5.49	$p < 0.0001$
	Diff of Mean:	-0.58	*Upper CI:*	-0.61	*Upper CI:*	-0.55	
VAT/m^3 [l/m^3]	0.75±0.42	0.03-3.45	0.61±0.36	0.03-2.84	0.90±0.43	0.05-3.45	$p < 0.0001$
	Diff of Mean:	-0.28	*Upper CI:*	-0.30	*Upper CI:*	-0.27	
%VAT*	33.6±12.7	6.2-70.5	23.8±6.5	6.2-53.4	44.3±8.4	17.3-70.5	$p < 0.0001$
	Diff of Mean:	-20.53	*Upper CI:*	-20.82	*Upper CI:*	-20.23	
VAT/TLT**	35.0±17.9	1.4-139.0	31.5±17.0	1.4-128.8	38.9±18.0	2.0-139.0	$p < 0.0001$
	Diff of Mean:	-7.39	*Upper CI:*	-8.09	*Upper CI:*	-6.70	
VAT/WEI	4.6±2.2	0.3-14.5	3.7±1.7	0.2-11.7	5.7±2.2	0.4-14.5	$p < 0.0001$
	Diff of Mean:	-2.00	*Upper CI:*	-2.07	*Upper CI:*	-1.92	

[#] = *Thigh Muscle Volume, * = VAT/TrunkAT, ** = VAT/Thigh Muscle Volume*

Table S3

a) Insulin sensitive (IS) vs. insulin resistant (IR) subjects in different age groups (AG) for the TDFS cohort

	females			males		
	AG1	AG2	AG3	AG1	AG2	AG3
age	19-37	38-51	52-77	18-39	40-54	55-75
n (IS/IR)	209/60	227/49	171/85	136/27	118/47	116/49
VAT [l] IS	1.66±0.96	2.44±1.28	3.34±1.62	3.48±2.06	5.21±1.96	5.94±2.07
VAT [l] IR	3.28±1.45	4.40±1.64	4.68±1.71	6.15±1.98	7.84±2.21	7.89±2.27
VAT/m [l/m] IS	0.99±0.57	1.46±0.76	2.03±0.99	1.91±1.12	2.91±1.07	3.36±1.17
VAT/m [l/m] IR	1.97±0.89	2.65±0.95	2.89±1.04	3.42±1.08	4.38±1.19	4.52±1.25
VAT/m² [l/m²] IS	0.59±0.34	0.88±0.45	1.24±0.61	1.05±0.61	1.63±0.59	1.90±0.66
VAT/m² [l/m²] IR	1.19±0.54	1.60±0.56	1.79±0.64	1.90±0.60	2.45±0.66	2.59±0.70
VAT/m³ [l/m³] IS	0.35±0.20	0.53±0.27	0.76±0.37	0.58±0.33	0.91±0.33	1.08±.0.38
VAT/m³ [l/m³] IR	0.71±0.33	0.97±0.34	1.11±0.40	1.06±0.34	1.37±0.37	1.49±0.40
%VAT IS	5.32±2.08	6.48±2.12	9.07±3.35	11.86±3.87	17.41±3.98	19.51±4.21
%VAT IR	7.00±2.40	8.82±2.21	10.66±2.64	13.89±4.37	17.55±3.82	20.64±4.01
VAT/TLT IS	3.96±1.91	5.55±2.56	8.06±3.56	6.06±3.18	8.87±2.96	10.70±3.35
VAT/TLT IR	7.33±2.98	9.16±2.90	10.96±3.39	9.43±2.66	12.52±2.93	13.68±3.34
VAT/WEI IS	2.12±0.90	2.88±1.16	4.07±1.63	3.67±1.69	5.45±1.56	6.40±1.76
VAT/WEI IR	3.49±1.28	4.44±1.22	5.30±1.40	5.37±1.49	6.97±1.41	7.76±1.73

b) healthy (H) vs. prediabetic (PRED) subjects in different age groups for the TDFS cohort

	females			males		
	AG1	AG2	AG3	AG1	AG2	AG3
age	19-37	38-51	52-77	18-39	40-54	55-75
n (H/PRED)	206/104	111/171	40/169	120/44	52/97	40/141
VAT [l] H	1.83±1.09	2.41±1.30	2.79±1.28	3.51±2.17	5.39±2.52	5.94±1.49
VAT [l] PRED	2.53±1.50	3.32±1.75	4.02±1.74	4.96±2.28	6.26±2.32	6.54±2.40
VAT/m [l/m] H	1.10±0.65	1.44±0.78	1.68±0.77	1.93±1.18	3.01±1.39	3.36±0.86
VAT/m [l/m] PRED	1.51±0.91	2.01±1.05	2.47±1.06	2.75±1.25	3.51±1.27	3.72±1.36

VAT/m² [l/m²] H	0.66±0.39	0.86±0.47	1.01±0.47	1.06±0.64	1.68±0.77	1.90±0.50
VAT/m² [l/m²] PRED	0.91±0.55	1.21±0.63	1.52±0.65	1.53±0.69	1.96±0.70	2.11±0.77
VAT/m³ [l/m³] H	0.39±0.24	0.52±0.29	0.61±0.28	0.58±0.35	0.94±0.43	1.08±0.30
VAT/m³ [l/m³] PRED	0.55±0.34	0.73±0.39	0.93±0.41	0.86±0.39	1.10±0.39	1.20±0.45
%VAT H	5.37±2.03	6.37±2.19	8.07±2.71	11.76±3.94	17.33±4.23	19.56±4.49
%VAT PRED	6.51±2.35	7.73±2.53	10.24±3.21	13.15±4.29	17.32±3.82	19.76±4.08
VAT/TLT H	4.30±2.13	5.44±2.67	6.61±2.78	6.04±3.22	8.94±3.36	13.98±2.92
VAT/TLT PRED	5.74±3.01	7.38±3.41	9.66±3.58	8.08±3.31	10.37±3.39	11.56±3.71
VAT/WEI H	2.25±0.96	2.81±1.21	3.47±1.36	3.66±1.73	5.45±1.72	6.42±1.59
VAT/WEI PRED	2.90±1.31	3.67±1.46	4.80±1.59	4.67±1.75	6.06±1.64	6.79±1.90

c) healthy (H) vs. prediabetic (PRED) subjects in different age groups in the UKBB study cohort

	females			males		
	AG1	AG2	AG3	AG1	AG2	AG3
age	40-51	52-59	60-70	40-52	53-60	63-70
n (H/PRED)	1653/112	1610/233	1267/311	1373/133	1402/270	1297/317
VAT [l] H	2.36±1.41	2.61±1.49	2.68±1.45	4.56±2.26	4.83±2.30	4.79±2.20
VAT [l] PRED	3.31±1.78	3.21±1.80	3.36±1.53	5.33±2.36	5.87±2.49	5.96±2.36
VAT/m [l/m] H	1.44±0.86	1.61±0.92	1.66±0.90	2.56±1.26	2.74±1.30	2.74±1.25
VAT/m [l/m] PRED	2.05±1.10	1.99±1.10	2.10±0.95	3.01±1.32	3.34±1.41	3.42±1.37
VAT/m² [l/m²] H	0.89±0.53	0.99±0.57	1.03±0.56	1.45±0.71	1.56±0.74	1.57±0.71
VAT/m² [l/m²] PRED	1.25±0.68	1.23±0.68	1.31±0.59	1.70±0.75	1.91±0.80	1.96±0.80
VAT/m³ [l/m³] H	0.54±0.33	0.61±0.36	0.64±0.35	0.82±0.40	0.89±0.42	0.90±.0.41
VAT/m³ [l/m³] PRED	0.77±0.43	0.76±0.42	0.81±0.37	0.97±0.42	1.09±0.47	1.13±0.47
%VAT H	21.8±5.9	23.7±6.2	25.2±6.7	42.3±8.1	44.4±8.2	45.1±8.4
%VAT PRED	24.0±6.3	25.2±6.6	27.3±6.8	43.2±9.0	46.6±7.9	47.6±8.5
VAT/TLT H	26.9±15.2	31.4±16.7	33.5±17.1	34.1±16.5	38.2±17.3	40.0±17.8
VAT/TLT PRED	36.1±18.2	37.6±19.4	41.5±17.8	40.3±17.5	46.7±19.1	49.8±19.8
VAT/WEI H	3.23±1.53	3.67±1.64	3.90±1.68	5.17±2.08	5.64±2.12	5.76±2.14
VAT/WEI PRED	4.10±1.78	4.22±1.80	4.66±1.70	5.88±2.14	6.54±2.14	6.83±2.23

Table S4

VAT-related indices for categorized variables of insulin sensitive (IS) and insulin resistant (IR) as well as healthy (H) and prediabetic (PRED) females and males

**Females**	n		VAT [l]	VAT/m [l/m]	VAT/m² [l/m²]	VAT/m³ [l/m³]	%VAT	VAT/TLT	VAT/WEI
**TDFS**									
	607	IS	2.44	1.47	0.89	**.053**	6.82	5.72	2.96
	194	IR	4.18	2.55	1.56	**0.95**	9.02	9.39	4.51
p			<0.0001	<0.0001	<0.0001	**<0.0001**	<0.0001	<0.0001	<0.0001
Diff of Mean			1.74	1.08	0.67	**0.42**	2.19	3.67	1.54
upper CI			2.00	1.24	0.77	**0.48**	2.69	4.21	1.80
lower CI			1.49	0.93	0.58	**0.36**	1.70	3.13	1.30
	357	H	2.12	1.27	0.76	0.46	5.97	4.90	2.55
	444	PRED	3.40	2.07	1.26	0.77	8.40	7.85	3.91
p			<0.0001	<0.0001	<0.0001	<0.0001	<0.0001	<0.0001	<0.0001
Diff of Mean			1.28	0.80	0.50	0.31	2.42	2.95	1.36
upper CI			1.50	0.93	0.58	0.36	2.82	3.41	1.57
lower CI			1.06	0.67	0.42	0.26	2.03	2.50	1.16
**UKBB**									
	4176	H	2.54	1.56	0.96	0.59	23.43	30.37	3.57
	598	PRED	3.34	2.07	1.28	0.80	26.23	39.60	4.45
p			<0.0001	<0.0001	<0.0001	<0.0001	<0.0001	<0.0001	<0.0001
Diff of Mean			-0.75	-0.48	-0.30	-0.19	-2.60	-8.72	-0.83
upper CI			-0.87	-0.55	-0.35	-0.22	-3.13	-10.10	-0.96
lower CI			-0.63	-0.40	-0.26	-0.16	-2.07	-7.33	-0.69

Males	n		VAT [l]	VAT/m [l/m]	VAT/m² [l/m²]	VAT/m³ [l/m³]	%VAT	VAT/TLT	VAT/WEI
**TDFS**									
	370	IS	4.69	2.62	1.47	0.82	15.89	8.24	5.01
	124	IR	7.37	4.15	2.34	1.32	17.94	12.11	6.86
p			<0.0001	<0.0001	<0.0001	<0.0001	<0.0001	<0.0001	<0.0001
Diff of Mean			2.68	1.53	0.87	0.50	2.05	3.87	1.85
upper CI			3.13	1.78	1.01	0.58	3.06	4.59	2.24
lower CI			2.23	1.28	0.73	0.42	1.04	3.15	1.47
	212	H	4.41	2.45	1.36	0.76	14.52	7.58	4.59
	282	PRED	6.21	3.50	1.97	1.12	17.92	10.63	6.22
p			<0.0001	<0.0001	<0.0001	<0.0001	<0.0001	<0.0001	<0.0001
Diff of Mean			1.80	1.05	0.61	0.36	3.40	3.05	1.63
upper CI			2.24	1.29	0.75	0.43	4.28	3.72	1.99
lower CI			1.37	0.81	0.48	0.28	2.53	2.38	1.28
**UKBB**									
	4071	H	4.72	2.68	1.52	0.87	43.89	37.41	5.52
	720	PRED	5.83	3.33	1.90	1.09	46.51	47.03	6.56
p			<0.0001	<0.0001	<0.0001	<0.0001	<0.0001	<0.0001	<0.0001
Diff of Mean			-1.11	-0.65	-0.38	-0.22	-2.62	-9.62	-1.04
upper CI			-1.29	-0.75	-0.44	-0.26	-3.28	-11.04	-1.21
lower CI			-0.93	-0.55	-0.32	-0.19	-1.96	-8.19	-0.87

Data are presented as Mean with Difference of Mean (Diff of Mean) and upper/lower 95%-confidence intervals (CI). Where VAT/m = VAT/body height, VAT/m² = VAT/body height², VAT/m³ = VAT/body height³. The UKBB MRI protocol does not include measurement of total adipose or lean tissue, therefore within this dataset %VAT = VAT/total abdominal adipose tissue, VAT/TLT = VAT/thigh muscle volume.

Figure S1

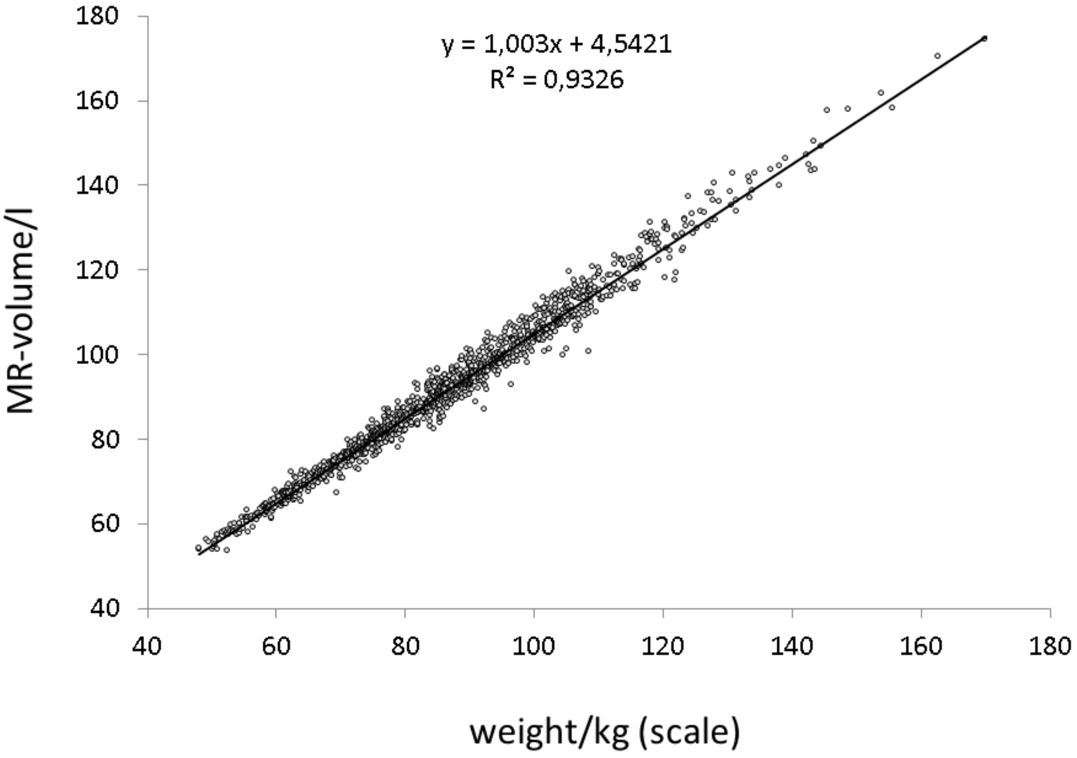

Figure S1: *Scatter plot of body weight vs. MR-derived volume after correction for fat density and inclusion of bone mass*
